# A new synthetic lure for management of the invasive giant African snail, *Lissachatina fulica*

**DOI:** 10.1371/journal.pone.0224270

**Published:** 2019-10-29

**Authors:** Amy Roda, Jocelyn G. Millar, Chris Jacobsen, Robin Veasey, Lenny Fujimoto, Arnold Hara, Rory J. McDonnell

**Affiliations:** 1 Animal Plant Health Inspection Service, Plant Protection and Quarantine, United States Department of Agriculture, Miami, Florida, United States of America; 2 Department of Entomology, University of California, Riverside, Riverside, California United States of America; 3 Department of Plant and Environmental Protection Services, University of Hawaii, Hilo, Hawaii, United States of America; 4 Department of Crop and Soil Science, Oregon State University, Corvallis, Oregon, United States of America; University of Minnesota, UNITED STATES

## Abstract

Synthetic chemical lures mimicking pheromones or food attractants are essential tools in eradication programs for invasive species. However, their uses in programs aiming to control or eradicate terrestrial gastropods are largely unexplored. The goal of this study was to find a synthetic attractant that could aid in the eradication or management of the giant African snail (*Lissachatina fulica*). Field studies in Hawaii showed that a commercial papaya-flavored oil attracted snails. Analysis of the odor profile of the oil identified a total of 22 chemicals, which comprised > 98% of the volatile compounds emitted by the oil. A synthetic blend was reconstructed that mirrored the release rates of the papaya oil odors. In laboratory and field bioassays, the reconstructed blend, applied to cotton wicks as water and canola oil or water and mineral emulsions, attracted more snails than the water and oil emulsion control wicks. Field studies in Hawaii and Florida showed that the reconstructed blend in an oil emulsion was not attractive to non-target species such as butterflies or bees. The snails were attracted from distances > 1 m and entered traps baited with the attractant emulsion. When tested in the South Florida giant African snail eradication program, direct ground application of the reconstructed papaya-flavored oil emulsion increased the number of snails killed by over 87% compared to water emulsion controls. Integrating tactics using the synthetic papaya oil attractant into control measures should increase the effectiveness of eradication and management programs.

## Introduction

Semiochemically-based lures are widely used in insect detection, management, and eradication programs worldwide [1, 2]. Some are based on pheromones, which generally impart a high degree of species-specificity, whereas others are based on food- or host-plant attractants, and may be somewhat less species-specific. However, by manipulation of components and blend ratios, preferential attraction of a given target insect species is usually possible. In contrast, analogous lures targeting pest slug and snail species have not yet been developed, although a number of food-based baits have been commercialized for management of terrestrial gastropod species [3].

Gastropod management programs typically employ chemical (molluscicides) and cultural (hand collection and debris removal) tactics [3]. However, slug and snail control programs can be hampered by the biological characteristics of the target species. For example, gastropods have a limited foraging range [4], and they may shelter in protected areas that might not receive sufficient rates of pesticide to kill the target animals. In addition, molluscicides such as metaldehyde are toxic to vertebrates [5], and are water soluble and so dissipate rapidly in humid climates [6]. The effectiveness of control strategies could be enhanced if an activator and/or attractant were used to increase snail movement, and to draw them to traps (mass trapping) or to a killing agent (attract and kill), or to exposed areas so that they could be killed or removed. Attractant-based methods would also help to draw pests away from areas where pesticides cannot be used, such as organic farms, and areas where children, pets, or farm animals could come in contact with toxicants.

Gastropods feed on a wide range of plant and animal materials, and a variety of substrates have been tested as components of baits [7–13]. However, using such materials as gastropod attractants in management programs is not without problems. For example, attraction to a food source may change with the age, ripeness, or stage of decay of the plant material [14] and must compete effectively with the odors from crops or other sources of attractants [7]. Additionally, rotting plant material may attract maggots, rodents, or other vermin [15]. For these and other reasons, the development of gastropod attractants based on reconstructions of food-based odors from synthetic chemicals would have a number of advantages. For example, the formulation and release rates of synthetic lures could be easily standardized, they could be made in any desired quantities, and release devices could be readily stored and shipped, analogous to the numerous semiochemically-based lures that are in routine use for insect pests. Furthermore, it may be possible to develop synthetic formulations that are more target-specific than crude attractants based on fresh or fermenting food-based materials.

Giant African snails (*Lissachatina fulica* (Férussac, 1821)), herein referred to as GAS, are considered among the world’s worst invasive pests, because they feed on a wide variety of agricultural crops and native plants [16]. The snails have been widely dispersed outside their native range by international commerce, including deliberate human-mediated movement in order to exploit GAS as a food source [17]. They have also been introduced accidently or illegally into new areas of the world, resulting in costly eradication or containment programs [18]. GAS also represent a significant threat to human health by acting as intermediate hosts of the rat lungworm (*Angiostrongylus cantonensis*) [19]. Humans become infected by ingesting raw produce contaminated with gastropod slime containing *A*. *cantonensis* larvae, or by eating infected raw or undercooked gastropods. The immature worms migrate to the human brain, causing the often fatal disease eosinophilic meningoencephalitis [20, 21].

We report here the results of our studies on developing a synthetic attractant for GAS, based on a commercially available papaya-flavored oil. During the course of the project, we discovered that the test formulations also attracted another invasive gastropod, the semi-slug *Parmarion martensi*, which is also an important vector of the rat lungworm nematode [22].

## Materials and methods

### Field locations

In Florida, properties were located within quarantine zones established by the United States Department of Agriculture and Florida Department of Agriculture and Consumer Services Giant African Land Snail joint eradication program. Property owners within the cores signed release forms granting full permission for accessing and conducting control efforts. In Hawaii, the study was conducted at the Komohana Research and Extension Center (KREC) and permission was granted by the University of Hawaii at Hilo. In Trinidad, the field location was within giant African snail control area and permission was granted by The Ministry of Agriculture, Land and Marine Resources. There were no endangered or protected gastropod species present in any of the field study sites.

### Field bioassays with fruit-flavored oils

Papaya and banana fruit [15, 23, 24] and food extracts [25] have been shown to draw GAS into traps, suggesting that the odors from these substrates attracted the snails. Commercially available papaya- and banana- flavored oils (Cat. # NF-3763/NAT and NF-3763/NAT respectively; Natures Flavors, Orange, CA, USA) were tested to determine whether GAS moving toward daytime refuges would orient towards and feed on lures dosed with one of the flavored oils. Lures were made by saturating standard sized cotton dental wicks (Patterson Dental Supply, St. Paul MN, USA) with 4 ml of an emulsion prepared from a 1:1:0.2 mixture of tap water, mineral oil, and Tween®80 surfactant. The flavored oil (200 ul) was then pipetted onto each emulsion-saturated wick (4200 ul total volume). The wicks were positioned in the center of 60 mm diameter polystyrene Petri dishes (Fisherbrand^TM^) and then were placed near (25–50 cm) from foraging snails at KREC (19.696029N, 155.089564W). The site had established populations of invasive gastropods and no management efforts were undertaken during the periods of experimentation. Ten replicates were placed 2–5 m apart, each with 1–6 GAS (ca.30-70 mm) in the vicinity. Each replicate consisted of three wicks, each dosed as above with one of three treatments: 1) papaya-flavored oil emulsion 2) banana-flavored oil emulsion, and 3) oil emulsion alone as a control. Within a replicate, the three treated dishes were randomly spaced 50 cm apart. The treatments were observed continuously for 2 h, recording the number and size of GAS that changed direction towards a given treatment, and the number that fed on each wick.

### Analysis and reconstruction of the odor of the papaya-flavored oil

A sample of 2 ml of the papaya-flavored oil described above was pipetted into a 20 ml glass vial, which was then sealed with aluminum foil. The headspace odors were sampled by inserting a solid phase microextraction fiber (SPME, polydimethylsiloxane film, 100 μ film; Supelco, Bellefonte PA, USA) through the foil for 20 sec. The fiber was then thermally desorbed directly into an Agilent (Santa Clara CA, USA) 6890N gas chromatograph coupled to an Agilent 5975C mass selective detector. Fibers were desorbed in split-less injection mode. The injection port temperature was 250°C, and the fiber was desorbed for 20 sec before starting the temperature program. The GC was fitted with a 30 m x 0.25 mm ID DB-5 column (J&W Scientific, Folsom CA, USA), which was programmed from 40°C for 1 min, then 5°C per min to 250°C, hold at 250°C for 10 min. Helium was used as carrier gas with a linear flow of 37 cm/sec. Mass spectra were obtained in electron impact ionization mode (70 eV), with a scanning range of 30–450 daltons. Compounds were tentatively identified by matches with database spectra (NIST database) and/or interpretation of their spectra. Identifications were confirmed by matching their retention times and mass spectra with those of authentic standards, obtained from the sources shown in Table 1.

**Table 1:** Compounds identified from commercial papaya-flavored oil by solid phase microextraction followed by analysis by gas chromatography-mass spectrometry. Amounts are normalized to the most abundant compound, 3-methylbutyl acetate, in the oil.

Peak #	Compound	Relative amount from analysis	Corrected amount^a^	Chemical class subset^b^	Source of authentic standard^c^
**1**	Acetone	1.5	1.3	Ketones	Fisher
**2**	Ethyl acetate	22.3	12	Esters	Fisher
**3**	Acetoin	1.4	20	Ketones	TCI
**4**	3-Methylbutanol	1.0	1.5	Alcohols	Fisher
**5**	Ethyl butyrate	6.4	5.2	Esters	Spectrum
**6**	Ethyl 3-methylbutyrate	11	38	Esters	Acros
**7**	(*Z*)-3-Hexenol	44	38	Alcohols	Aldrich
**8**	Hexanol	1	2.5	Alcohols	Alfa-Aesar
**9**	3-Methylbutyl acetate	100	100	Esters	Acros
**10**	α-Pinene	48.3	92	Terpenes	Aldrich
**11**	Benzaldehyde	1.2	2.7	Ketones	Alfa-Aesar
**12**	β-Pinene	2.9	7.4	Terpenes	Aldrich
**13**	(*Z*)-3-hexenyl acetate	21.8	50	Esters	Sigma
**14**	Hexyl acetate	11	25	Esters	TCI
**15**	Limonene	9.8	27	Terpenes	Acros
**16**	Benzyl alcohol	2.3	26	Alcohols	Sigma
**17**	Benzyl acetate	0.14	1.7	Esters	Aldrich
**18**	Neral	0.41	23	Ketones	Acros
**19**	Geranial	0.47	21	Ketones	Acros
**20**	(*Z*)-3-hexenyl hexanoate	0.21	34	Esters	Sigma
**21**	Hexyl hexanoate	0.51	58	Esters	Aldrich
**22**	β-caryophyllene	0.30	26	Terpenes	TCI

^a^Corrected amount = relative volume to mix to create a blend with an odor profile matched to that of the commercial papaya flavored oil.

^b^Subsets of compounds grouped by chemical classes for bioassays.

^c^Fisher = Fisher Scientific (Pittsburg PA); TCI = TCI America (Portland OR); Spectrum = Spectrum Scientific, Irvine CA; Acros = Acros Organics,; Aldrich = Aldrich Chemical, Milwaukee WI; Alfa-Aesar, Ward Hill MA; Sigma = Sigma Chemical, St. Louis MO)

**Fig 1:**
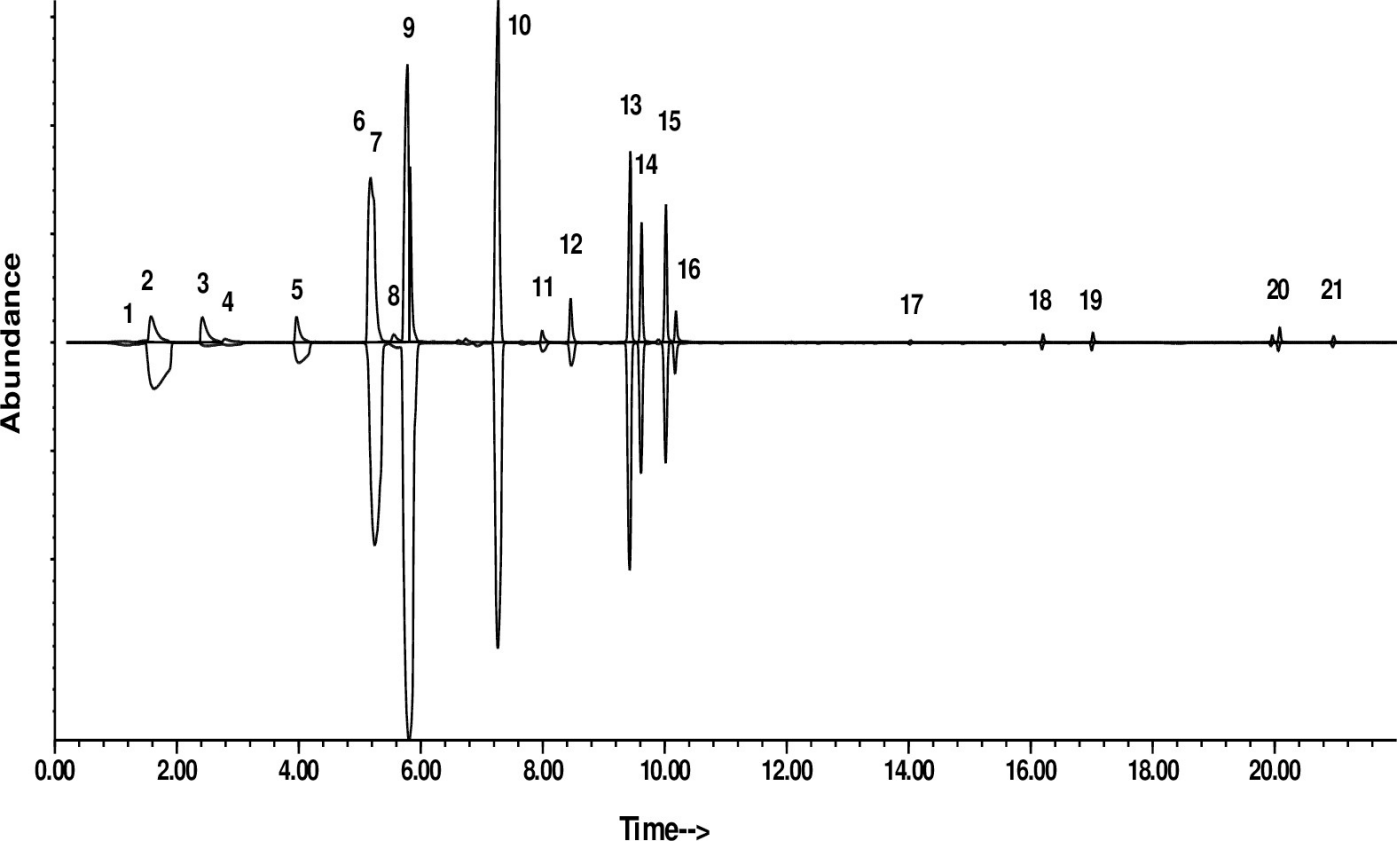
Top trace: Total ion chromatogram of reconstructed odor blend of the papaya-flavored oil in canola oil carrier; Inverted bottom trace: Total ion chromatogram of papaya-flavored oil as received from the supplier. Peak numbers correspond to the compounds listed in Table 1.

To reconstruct the odor profile, 0.1 ml aliquots of each of the 22 compounds listed in Table 1 were combined, and 0.1 ml of the resulting mixture was diluted in 10 ml of canola oil as a carrier. The headspace odors of a 100 μl aliquot were analyzed by SPME and GC-MS as described above, with the resulting ratios of peak areas providing the relative volatilities of the compounds, using the most abundant compound in the original analysis (3-methylbutyl acetate) as a standard. Thus, dividing the peak area of 3-methylbutyl acetate by the peak area of compound X provided the relative volatility. This value, multiplied by the relative amount of compound X compared to 3-methylbutyl acetate, which was arbitrarily set to 100, provided the corrected amount of each compound. These amounts were used to obtain a headspace profile approximately mimicking that of the original papaya-flavored oil (Fig 1). With these relative ratios determined, batches of the reconstituted odor blend were made up as needed, by mixing the appropriate volumes of the various components in the ratios shown in Table 1.

For laboratory and field trials, the odor blend was mixed into an oil and water emulsion which served as a slow-release formulation. For all subsequent laboratory and field trials, except stated otherwise, 44 μl (0.2% concentration) or 440 μl (2% concentration) of the reconstructed odor blend or commercial oil was added to 10 ml water, 2 ml Tween®80 surfactant and 10 ml of either canola oil (herein canola oil emulsion) or mineral oil (herein mineral oil emulsion). The resulting mixture was shaken vigorously to form an emulsion. Cotton dental wicks were soaked in the emulsion until saturated (approximately 4 ml per wick) with the exception of the laboratory bioassay done in Riverside California where 1 ml of the resulting emulsion was used.

### Laboratory bioassays

A colony of GAS was established in the Biosecurity Level II quarantine facility at the University of California Riverside, from wild snails collected in Miami, Florida (USDA Permit P526P-12-02858). Snails were held at 24°C and 50% RH, and were fed organic carrots. A piece of oyster shell cake (Down to Earth^TM^ Oyster Shell) mixed with water, was provided as a calcium source. This diet was supplemented weekly with dry cat food (Whiskas^TM^ brand) and bimonthly with organic cucumbers. The day before a bioassay, six snails were randomly selected and held in containers (5.5 l) and starved for 18 h. In a separate room (24°C, 50% RH, fluorescent lighting), bioassays were carried out in open-topped plastic box arenas (56 x 41 x14 cm). The bottom of each box was covered with a moistened paper towel. Cotton dental wicks dosed with 1 ml of treatment or control were placed on glass Petri dishes (10 cm) spaced 15 cm apart, 13 cm from the back wall of the box. The treatments used were prepared as described above. Six replicates (~55 cm apart) were run simultaneously with treatments and controls alternated between sides in neighboring arenas.

An individual snail was released in the center of the box between the 2 dishes, spritzed with deionized water, and allowed to move freely for up to 1 h. A choice was scored when they touched a dish. Any snail that failed to touch one of the dishes within 1 h, or that left the arena floor was recorded as a non-responder. After each bioassay, arenas were cleaned and rinsed with deionized water, the paper towel flooring was replaced, and the positions of treatments and controls were switched from the previous assay, with fresh dishes and wicks. Snails were used only once. Four two-choice experiments were conducted: 1) commercial papaya-flavored oil at 2% concentration in a 1:1:0.2 water, canola oil emulsion, and Tween®80 vs untreated emulsion (n = 12), 2) the synthetic reconstruction of the papaya-flavored oil at 2% concentration in the canola oil emulsion vs untreated emulsion (n = 24), 3) the synthetic reconstruction of papaya-flavored oil at 2% concentration in the emulsion vs the commercial papaya-flavored oil at 2% concentration in the canola oil emulsion (n = 24) and 4) a blank canola oil and water emulsion vs a blank mineral oil and water emulsion (n = 48). For the blank emulsion choice test, both adult (>40 mm shell length, n = 24) and immature (6–13 mm, n = 24) snails were used.

### Field studies with the synthetic lure

#### Comparison of the synthetic lure and commercial papaya-flavored oil attraction

The synthetic lure and commercial papaya-flavored oil were tested at KREC in Hilo, HI from May 21–25, 2016. Preliminary experiments showed that a 2% and 0.2% concentration of the neat reconstructed odor blend in the emulsions were equally attractive to GAS but 0.2% appeared to be more attractive to the invasive semi-slug, *Parmarion martensi* (McDonnell and Jacobsen, pers. obs.). Thus, lures were tested at 0.2% concentration mixed in a 1:1:0.2 canola oil, water, and Tween®80 emulsion. At dusk, an emulsion-soaked wick (4 ml/wick) was placed on a plastic Petri dish (9cm), which was then enclosed in a 3.75 oz (111 ml) net pot, which in turn was placed just inside a 19.7 x 19.7 x 31.8cm horticultural production container (Stuewe & Sons TP812; Tangent OR, USA). Metaldehyde (Deadline® 4%) was sprinkled inside and immediately adjacent to the production container at the recommended application rate (9 kg/4047 m^2^). The pot protected the lure from heavy rains and potential removal by non-target mammals while allowing the odor to disperse. The pots were placed 50 cm apart from each other and at ~1 m from a known population of GAS. One of three treatments (n = 10) were randomly assigned to each pot: 1) commercial papaya-flavored oil emulsion lure, 2) reconstructed papaya-flavored odor emulsion lure or 3) untreated emulsion as a control. After 12 h, all gastropods found in the pot and within 15 cm of the pot were collected, identified, and counted. The bioassay was run for four consecutive nights with a fresh wick and additional metaldehyde bait reapplied after heavy rain as needed. The cumulative number of each species of gastropods collected per treatment over the four nights was analyzed.

#### Attractant deployment methodologies

To test whether the synthetic lure could be directly combined with liquid metaldehyde (Slug-Fest, OR-CAL, Inc. Junction City, OR, USA), one of two spray treatments (250 ml) were applied to 50 x 50 cm plots: 1) liquid metaldehyde diluted with water (2.9 ml Slug-Fest/ 250 ml tap water) or 2) 0.2% synthetic lure emulsion (0.5 ml) mixed with the liquid metaldehyde. Plots (n = 50) were established at least 50 cm apart and at ~1 m from a known population of GAS at KREC. The species and number of gastropods were recorded and left in the plots 2 h after treatment application. After 18 and 48 h, all gastropods found in the plots were counted and removed.

To test whether the oil base used affected how long the lure remained attractive to GAS, we tested wicks treated with the synthetic blend formulated in either mineral oil or canola oil emulsions. In the same experiment, we tested fresh formulations versus formulations that had been aged outdoors for 48 h. As above, each oil was tested as a 0.2% synthetic lure emulsion. The wicks were aged outdoors under an overhang to shelter the wicks from rain but allow for sun and air exposure. After 48 h, 4 ml of liquid metaldehyde (1.15 ml Slug-Fest /100 ml tap water) was added to the aged wicks, to freshly prepared wicks treated with the 0.2% synthetic lure in the oil emulsions, and to dry wicks. The wicks of each of the 5 treatments: 1) 48 h aged synthetic lure in mineral oil emulsion 2) 48 h aged synthetic lure in a canola oil emulsion, 3) fresh synthetic lure in mineral oil emulsion, 4) fresh synthetic lure in a canola oil emulsion and 5) untreated control, were placed at dusk 75 cm apart in plastic Petri dishes, 1 m from areas where foraging snails were observed 12 h earlier (n = 50) at KREC. After 12 h, all gastropods found within 15 cm of the test treatments or controls were counted, identified, and collected.

To determine if the synthetic lure drew GAS into traps, Snailer Snail and Slug Traps^TM^ (American Organic Products, Ventura CA, USA) were baited with cotton wicks dosed with one of three treatments: 1) liquid metaldehyde (1.15 ml Slug-Fest /100 ml tap water) alone, 2) liquid metaldehyde and synthetic lure (0.2%) in canola oil emulsion, and 3) liquid metaldehyde and synthetic lure (0.2%) in mineral oil emulsion. Three replicates of the baited traps were placed at dusk in areas at KREC where foraging GAS were observed 12 h earlier. After 12 h, the traps were inspected and all gastropods counted and removed. The traps were cleaned and the lures replaced. The traps were moved daily to three other locations where GAS had been observed, and the collection, cleaning, re-baiting and moving of traps procedure was repeated, to provide a total of 12 spatial and temporal replicates

#### Snail behavioral observations

Behavioral studies to determine the draw distance and the length of time that GAS fed on the lures were conducted at a second study site in Hilo, Hawaii (19.71666N, 155.07838W), adjacent to an infested hibiscus hedge. The site had a large mixed age population of GAS and no control measures had been conducted. Petri dishes (n = 15) were baited with one of 4 treatments: 1) 48 h aged synthetic lure in mineral oil emulsion 2) 48 h aged synthetic lure in canola oil emulsion, 3) fresh synthetic lure in mineral oil emulsion and 4) fresh synthetic lure in canola oil emulsion. The baited dishes were placed at 20:20 h in groups of four, 50 cm apart and 50 cm from the hedge. The 0.2% synthetic lure and oil emulsions were as described above. The wicks were continuously monitored for 2 hr. All snails touching the wicks were counted, measured, and marked with fluorescent nail polish. The time when the snails first touched and when the snails left the wicks were recorded. When movement toward the lure was observed with snails >25 cm from the dish, the original position of the snail was marked and the distance to the Petri dish was measured once the snail touched the dish.

#### Non-target studies

To determine if the synthetic lure attracted beneficial insects and other non-target animals, studies were conducted on a residential property in Miami, FL (25.405852N, 80.526497W) and Hawaii, HI at two locations, Umauma (19.915911, -155.140856) and Mountain View (19.643813, -155.079941). In Miami, cotton wicks saturated with 4 ml of the 0.2% synthetic lure in mineral oil emulsion were placed in the center of white sticky trap liners designed for catching insects (Great Lakes IPM, Inc., Vestaburg MI, USA). Controls consisted of wicks treated with emulsion only, and dry wicks. The traps (n = 6) were placed on the ground at 14:00 h during peak insect activity near areas where bees and butterflies were seen foraging. The traps were observed continuously for 1 h after placement and then every hour until dusk. Any insect or other non-target activity such as hovering or landing on or near the wick was recorded. In Hawaii, cotton wicks saturated with 0.2% synthetic lure in canola oil emulsion or untreated emulsion were placed atop 12.7 x 17.8 cm sticky cards inside Delta Traps (Great Lakes IPM). At sunset, the traps (n = 4 at each of the two sites) were placed horizontally at ground level or hung 1.5 m above ground level. After 24 h, the traps were collected in both locations and examined primarily for honey bees, native bees, and butterflies, but other insect taxa were also recorded.

#### Testing the synthetic lure in GAS eradication programs

In Trinidad, a study was conducted in a combined residential and small farm community (10.644002N, 61.437157W) in a roadside area (100 m x 25 m) that contained a large number of GAS. Fifty plastic Petri dishes with a metaldehyde-soaked wick (1.15 ml Slug-Fest/100 ml of tap water) were placed 1 m apart and 1 m from foraging snails seen along the road at sunset. A second cotton wick soaked with 0.2% synthetic lure in canola oil emulsion was added to half the dishes (n = 25). After 12 h, all snails found within 25 cm of the dishes were measured and counted.

In Florida, a field bioassay was conducted to determine whether the synthetic odor lures might draw snails to the metaldehyde applied as part of the Florida GAS eradication program standard operating procedures. The study took place on a residential property in Miami (25.78252N, 80.23563W) 12 h after GAS were initially detected. The property was located in a quarantine zone established by the United States Department of Agriculture (USDA) and Florida Department of Agriculture and Consumer Services (FDACS) GAS joint eradication program. Upon discovery of GAS, the property underwent the standard eradication program procedures of debris removal followed by labeled rate (30 ml/3.8 l) application of liquid metaldehyde (Slug-Fest) in areas where snails were collected. Metaldehyde granular bait (Deadline^TM^) was broadcast in the remaining lawn areas of the property. Four ml of 0.2% lure in mineral oil emulsion or emulsion alone were pipetted directly on the ground as spot treatments in pairs (n = 110) within the liquid metaldehyde-treated areas on the infested property. Ground applications of the lure were made rather than dispersing wicks because debris removal conducted as part of normal eradication procedures would have removed most of the wicks. Lure applications were made at dusk near areas that had debris or dense vegetation or were hard to access (e.g. under porches), with the purpose of activating and attracting hidden snails to the pesticide-treated areas. The spot treatments were made 75 cm apart and marked with a flag, and all snails found within 15 cm of a treatment were measured and counted 12 h after application of the lure or control.

### Statistical analysis

Count data were tested for normality (Shapiro-Wilk test) and homoscedasticity (Bartlett test) using software from SAS Institute (Cary NC, USA [26]). The data that did not meet the assumptions for parametric analysis after transformation were analyzed with non-parametric tests (Mann-Whitney test and Kruskal-Wallis test [27]). Kruskal–Wallis tests were followed by a post-hoc test of multiple comparisons of all treatments [28]. The choice study data were analyzed using a binomial test [29]. *P-*values ≤ 0.05 were considered statistically significant.

## Results

### Field bioassays with fruit-flavored oils

Wicks treated with the papaya- and banana-flavored oil emulsions caused GAS to stop movement toward daytime refuge areas and return to the open. A total of 14 snails were observed changing direction and moving towards the lures. Of the 14 snails observed, more fed on wicks treated with the papaya-flavored oil (10 snails) than the banana-flavored oil (3 snails) or the mineral oil emulsion alone (1 snail). All snails observed were adult size class (50–80 mm).

### Analysis and reconstruction of the odor of the papaya-flavored oil

A total of 22 compounds were identified in the headspace odors of the commercial papaya-flavored oil (Table 1), comprising >98% of the total odor blend. The compounds included esters, ketones, aldehydes, alcohols, and terpenoids. Compounds were tentatively identified by matches of their mass spectra with database spectra (NIST 11 database) and/or interpretation of their spectra. Identifications were then confirmed by matching the retention times and mass spectra of the compounds with those of authentic standards. The 22-component odor blend was reconstructed from synthetic flavor and fragrance compounds (Fig 1), and the neat blend of compounds was diluted in either mineral or canola oil emulsions for laboratory and field testing.

### Laboratory bioassays

Snails that did not make a choice were discarded from the analyses. In two-choice arena bioassays, significantly more snails selected the commercial papaya-flavored oil emulsion over the untreated emulsion control (Table 2, Binomial test: *P =* 0.021). Similarly, more snails were attracted to the emulsion blended with the synthetic reconstruction of the papaya-flavored oil odors than to the control emulsion (Binomial test: *P* = 0.001). Although twice as many test snails selected the synthetic lure compared to the commercial oil lure (Table 2), this difference was not statistically different (Binomial test: *P =* 0.302). With the latter, a large number of snails made no choice, perhaps due to the competing odors of the two attractants. Both adult (> 40 mm) and immature (6–13 mm) snails showed no difference in attraction to the canola oil or mineral oil emulsions (Binomial test: *P =* 0.839). The same applied when the two age groups were lumped (Binomial test: *P =* 0.665).

**Table 2:** The percentage of *Lissachatina fulica* that selected various test treatments and controls, or made no choice, in two-choice laboratory bioassays testing emulsions of commercial papaya-flavored oil, synthetic reconstructions of the papaya oil odor in canola oil or mineral oil, or water emulsion controls.

Test treatments and controls	n	Treatment	Control	No Choice
Papaya flavored oil ^a^ emulsion /Untreated emulsion ^b^	12	75%	8.3%	16.7%
Synthetic lure emulsion/Untreated emulsion ^b^	24	75%	12.5%	12.5%
Synthetic lure emulsion/Papaya flavored oil emulsion ^b^	24	42%	21%	37%
Mineral oil + water emulsion/ Canola oil + water emulsion: Adult snails ^c^	24	54.2%	45.8%	0%
Mineral oil + water emulsion/ Canola oil + water emulsion: Immature snails ^c^	24	54.2%	45.8%	0%

^a^ Nature’s Flavors^®^ Natural Papaya Flavor Oil with sunflower oil and natural flavors as the listed ingredients

^b^ Emulsion formulations: 2% blend of reconstructed papaya-flavored oil odors or commercial oil in a 1:1:0.2 emulsion of water, canola oil, and Tween®80 surfactant; control was untreated emulsion

^c^ Adult snails > 40 mm, immature snails 6–13 mm

### Field studies with the synthetic lure

#### Comparison of synthetic lure and commercial papaya-flavored oil

**Fig 2:**
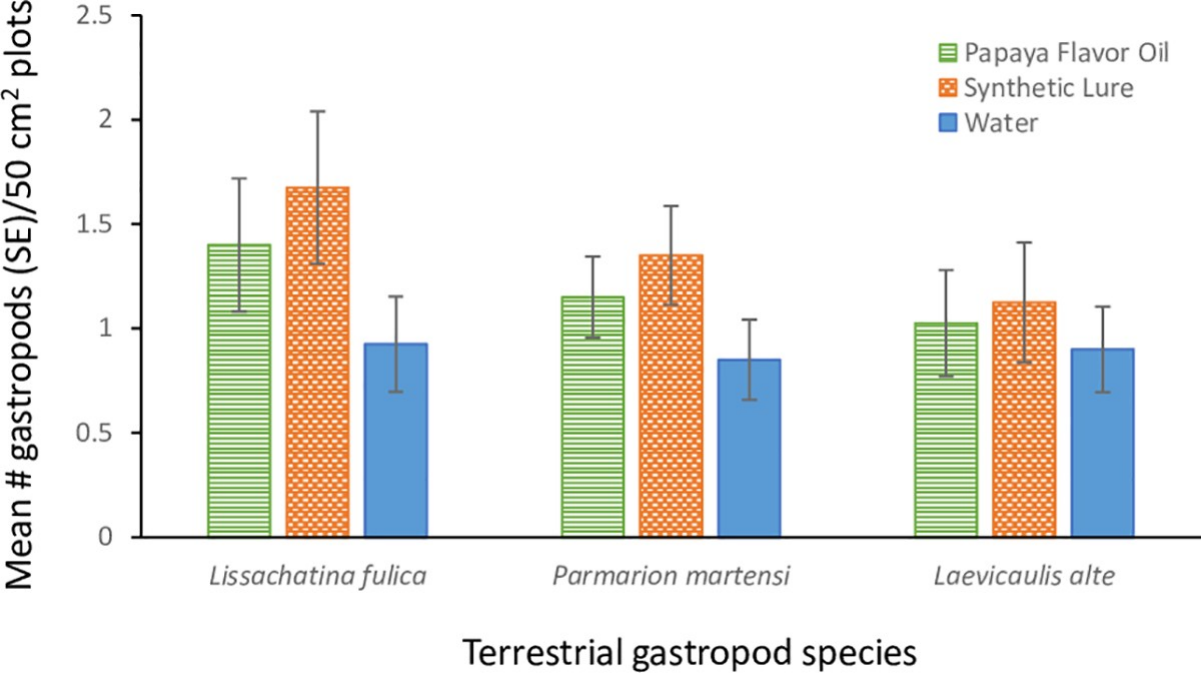
The mean numbers of giant African snails (*Lissachatina fulica*), semi-slugs (*Parmarion martensi*), and black slugs (*Laevicaulis alte*) found inside and adjacent to horticultural containers (n = 10) baited with metaldehyde (Deadline® 4% metaldehyde) and a canola oil emulsion containing 0.2% papaya-flavored oil (Nature’s Flavors®), or untreated emulsion. Bars represent one standard error.

Adding the commercial papaya flavored oil lure or the synthetic lure increased the number of GAS found in metaldehyde bait treated plots by 34 and 50% respectively compared to the number found in the control plots 12 h after application (Kruskal-Wallis test: X^2^ = 5.97 (2), P = 0.051; Fig 2). The numbers of black slugs (*Laevicaulis alte*) and semislugs (*P*. *martensi*) found in treatment and control plots were not significantly different (X^2^ = 0.34 (2), P = 0.82 and X^2^ = 3.26 (2), P = 0.20 respectively). There were no differences in the mean number of GAS collected from plots with the commercial papaya-flavored oil or the synthetic lure (z = 0.98, P = 0.33).

#### Attractant deployment methodologies

**Fig 3:**
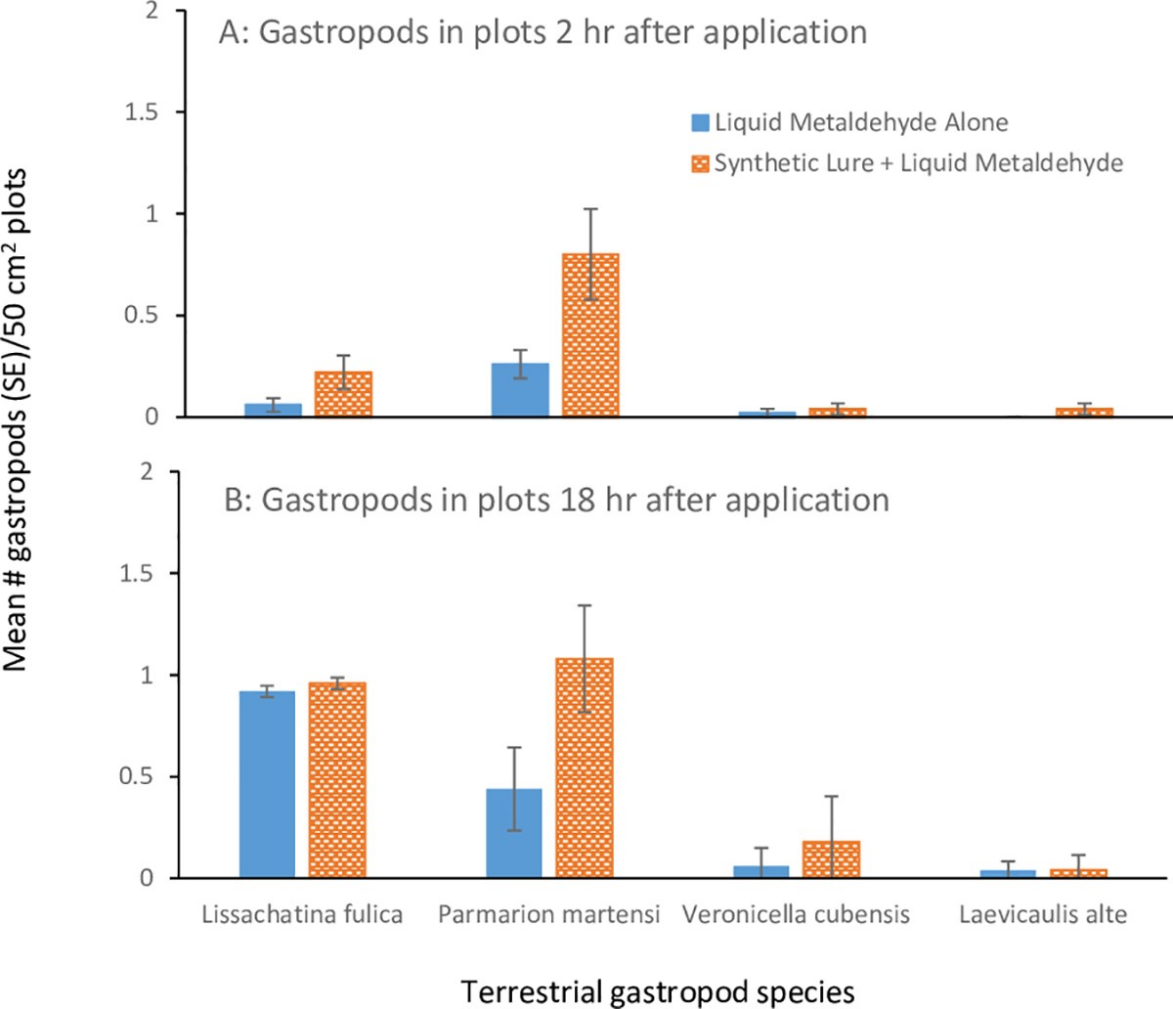
The mean number of giant African snails (*Lissachatina fulica*), semi-slugs (*Parmarion martensi*), Cuban slug (*Veronicella cubensis*) and black slugs (*Laevicaulis alte*) found in 50 cm^2^ plots (n = 50) 2 h (a) and 12 h (b) after application of liquid metaldehyde alone or liquid metaldehyde combined with 0.2% synthetic lure emulsion.

The attractiveness of the synthetic lure dissipated rapidly when mixed directly with liquid metaldehyde (Fig 3). Two h after application, significantly more GAS were found in with synthetic lure compared to the control plots (Unequal Variance (Welch) t test: t = 1.76 (65), p>t = 0.034; Fig 3A), but by 18 h after application there were no differences between the treatment and control plots (t = 0.12 (92), p>t = 0.44; Fig 3B). However, more *P*. *martensi* were found in plots with synthetic lure both 2 h (t = 2.32 (58), p>t = 0.01; Fig 3A) and 18 h (t = 2.66 (64), p>t = 0.005; Fig 3B) after application. After 48 h, only 5 additional GAS were found in the plots (2 in metaldehyde alone and 3 in synthetic lure/metaldehyde treatment). Nine additional semi-slugs were found in plots (2 in metaldehyde alone and 7 in synthetic lure/metaldehyde treatment). Adding the synthetic lure directly to liquid metaldehyde did not increase the number of *V*. *cubensis* or *L*. *alte* at any time period.

**Fig 4:**
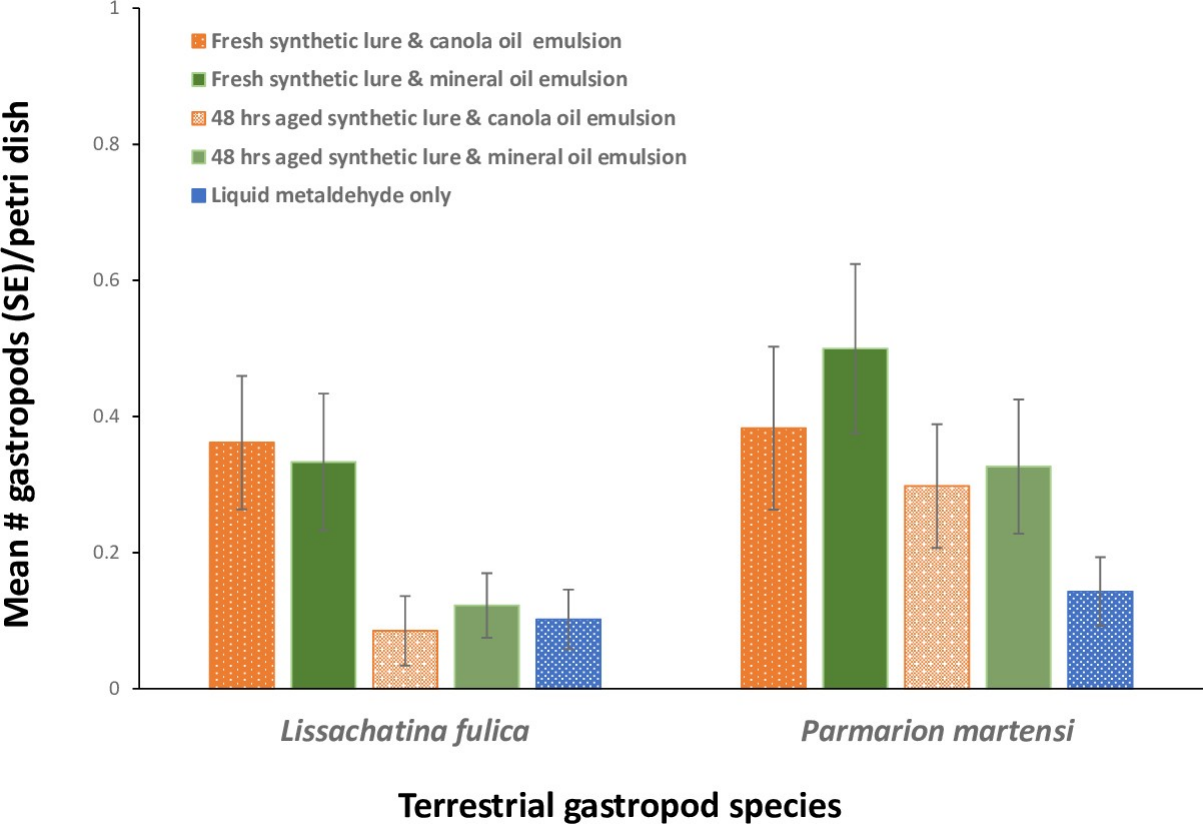
The mean number of giant African snails (*Lissachatina fulica*) and semi-slugs (*Parmarion martensi*) found in or near (15 cm) Petri dishes baited with wicks saturated with fresh or 48 h aged synthetic papaya odor lure (0.2%) combined with either mineral or canola oil and fresh liquid metaldehyde emulsion or liquid metaldehyde alone. Bars represent one standard error of the mean. Emulsions were 1:1:0.2 water/oil/ Tween®80.

When the synthetic lure emulsions were added to cotton wicks dosed with metaldehyde, the effects of random movement of gastropods into the plots was largely eliminated because the snails needed to contact the wick in order to receive a lethal dose of metaldehyde and a significant treatment effect was seen (X^2^ = 13.13 (4), P = 0.011). Significantly more GAS were found on or near wicks with synthetic lure mineral oil emulsion (z = 2.04, P = 0.04) or canola oil emulsion (z = 2.25, P = 0.02) compared to metaldehyde alone (Fig 4). There were no differences in the type of base oil (mineral or canola) used (z = 0.18, P = 0.86). For GAS, the attractive effect dissipated with time, as there were no differences between the number of GAS collected from the 48 h aged mineral oil (z = 0.31, P = 0.76) and canola oil (z = 0.63, P = 0.53) treated emulsions compared to metaldehyde alone. A number of wicks were removed during the experiment, likely by rodents seen in the area. Although wicks were lost from all treatments more were missing from the fresh lure in a mineral oil emulsion (8) than fresh lure in a canola oil emulsion (3), aged lure in a mineral oil emulsion (1), and aged lure in a canola oil emulsion (3).

**Fig 5:**
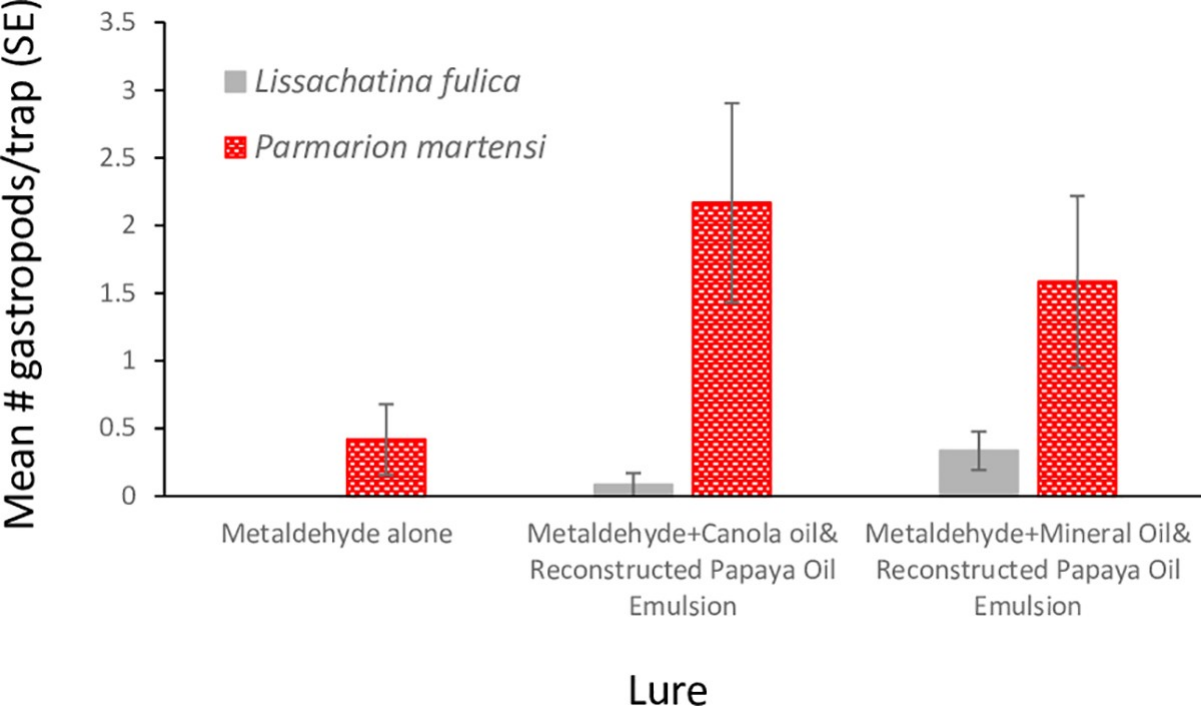
The mean number of giant African snails (*Lissachatina fulica*) and semi-slugs (*Parmarion martensi*) caught in Snailer Snail and Slug Traps^TM^ (n = 12) baited with liquid metaldehyde (1.15 ml in 100 ml of water) saturated cotton wicks, liquid metaldehyde combined with 0.2% synthetic lure + canola oil emulsion, or liquid metaldehyde combined with 0.2% synthetic lure + mineral oil emulsion. Bars indicate one standard error. Emulsions were 1:1:0.2 water/oil/ Tween®80.

Snailer traps baited with the synthetic emulsion lures with metaldehyde caught significantly more GAS (Fig 5; X^2^ = 5.87 (2), P = 0.05) than the traps baited with metaldehyde alone, which caught no snails. There were no significant differences between the numbers of GAS found in traps when the lure was formulated in either the mineral oil or the canola oil emulsions (z = 1.4, P = 0.15). The traps with the lure emulsions had 74–81% more *P*. *martensi* than traps containing only metaldehyde but the difference was not significant (X^2^ = 4.19 (2), P = 0.123, Fig 5). No *V*. *cubensis* or *L*. *alte* were found in any of the traps.

#### Snail behavioral observations

A total of 56 GAS were observed feeding on the wicks deployed near the hibiscus hedge from 20:40–22:40 (Table 3). No other gastropods were seen in the area. More wicks with fresh lure in canola oil emulsion attracted snails (60%), compared to the fresh lures in mineral oil emulsion or the 48 h aged lures in canola and mineral oil emulsions (40%, 20%, and 20% respectively). Although snails fed on the aged wicks, the number of snails observed decreased by 90% for the aged synthetic lure in canola oil and by 43% for the aged lure formulated in mineral oil. All treatments had GAS feeding on the wick within 20–30 min after placement. Feeding bouts lasted from ~1 min to > 1.25 h. All age classes fed on fresh lure emulsions and adult and juvenile snails fed on the aged lure emulsions. The initial locations of 5 snails that crawled > 25 cm directly to a fresh lure were recorded. A fresh synthetic lure in canola oil drew a snail > 105 cm from its original foraging location, which was the longest observed distance. Three additional snails were observed crawling towards fresh canola oil emulsion lures 35, 50, and 55 cm from the dish, and one snail was observed 50 cm from a fresh lure in a mineral oil emulsion before the snails were seen feeding on the lure. All other snails were first observed when they were ≤ 25 cm (43 snails) from the dish or feeding on the wick (8 snails).

**Table 3:** The number of individual giant African snails (*Lissachatina fulica*) observed feeding on wicks saturated with fresh and aged (48h) synthetic lure (0.2%) in a canola or mineral oil emulsion. Emulsions were 1:1:0.2 water/oil/Tween®80.

Treatment	# Adults (> 45 mm)	# Juveniles (20–45 mm)	# Neonates (< 20 mm)	Total # Snails	Mean(SE)/ snails/dish	Percentage of wicks with snails	Mean (SE) Feeding Duration (min)	Feeding Duration (min)
Fresh synthetic lure mineral oil emulsion	4	7	3	14	0.88 (0.23)	40	20 (4.11)	5–60
Fresh synthetic lure canola oil emulsion	5	24	1	30	1.73 (0.44)	60	25 (7.36)	10–45
48 h Synthetic lure mineral oil emulsion	3	5	1	9	0.66 (0.17)	20	18 (5.14)	1–50
48 h Synthetic lure canola oil emulsion	1	2	0	3	0.20 (.05)	20	40 (18.03)	15–75

#### Non-target studies

In the Miami non-target study, no bees or butterflies approached the wicks with the synthetic lure emulsion or the control emulsion despite numerous bees (*Apis mellifera*) and butterflies (Lycaenidae, Hesperiidae, Heliconiidae, and Nymphalidae) foraging in nearby flowers. Dogs present on the site did not approach the wicks or traps during the 24 h study. No differences were found in the insect composition on the sticky traps (Table 4). Insects trapped by random movement included flies, ants, leafhoppers, and thrips. No lures were removed by nontarget vertebrates, including reptiles (*Anolis sagrei*, *Basiliscus vittatus*, and *Coluber constrictor paludicola*) and amphibians (*Rhinella marina*) seen during the study. In Hilo, HI, the traps were not disturbed by vertebrate animals during the evaluation, foraging bees were not observed approaching the lures or the controls, and no bees were trapped. No differences were found in the number of insects on traps with the synthetic lure versus the controls. (Table 5).

**Table 4:** The mean number (SE) of insects arthropods found on sticky traps (n = 6) baited with a dry cotton wick (dry wick), a saturated wick with synthetic papaya oil lure in mineral oil emulsion (synthetic papaya lure emulsion) or a wick saturated with mineral oil and water emulsion (water emulsion) collected over 24 h in Miami, Florida, USA. Emulsions were 1:1:0.2 water/oil/ Tween®80.

Attractant	Diptera	Hemiptera	Collembola	Hymenoptera	Thysanoptera	Coleoptera
Dry Wick	83 (12.9)	36.2 (7.4)	122.6 (18.0)	9.4 (0.9)	2.8 (1.2)	0.4 (0.2)
Synthetic Papaya Lure Emulsion	80.5 (11.8)	33.25 (6.75)	123.75 (40.7)	12.25 (0.6)	2.25 (0.6)	0.75 (0.5)
Water Emulsion	96.25 (16.8)	36.25 (3.8)	127.5 (34.2)	11.25 (2.7)	2.75 (1.1)	0.5 (0.3)

**Table 5:** The pooled mean numbers (SE) and types of insects captured (indicated by ‘+’) on sticky cards (n = 8) baited with cotton wicks saturated with water, canola oil emulsion, synthetic papaya oil lure (0.2%) in water or synthetic papaya oil lure (0.2%) in a canola oil emulsion collected over 24 h in Hilo, HI, USA. Sticky card were suspended 1m (elevated) or placed on the ground (ground). Emulsions were 1:1:0.2 water/oil/ Tween®80.

Insects	Elevated				Ground			
	Water	Canola Oil Emulsion	Water + Synthetic Lure	Water + Synthetic Lure Emulsion	Water	Canola Oil Emulsion	Water + Synthetic Lure	Water + Synthetic Lure Emulsion
Mean (SE) All Insects/Card	2.25 (1.03)	2.25 (1.31)	3.25 (0.85)	2.75 (0.63)	10.25 (6.06)	7.75 (1.93)	13.75 (6.93)	13.50 (4.79)
Aranae	+			+	+			
Amphipoda					+			+
Anisopidae	+			+		+		
Anthocoridae				+				
Aphididae	+							+
Blattidae,					+			
Cecidiomyidae	+		+			+		
Ceratopogonidae			+					
Cercopidae						+		
Chironomidae		+		+	+		+	+
Cixiidae								+
Delphacidae								+
Entomobryidae					+	+	+	+
Eulophidae		+		+				
Formicidae		+						+
Lepidoptera (micro)		+	+					
Myridae					+			
Phoridae				+	+	+	+	+
Psocoptera			+		+			
Psychodidae	+					+		
Psyllidae		+		+		+		
Pulicidae								+
Sciaridae	+	+	+	+		+	+	+
Scatopsidae					+			
Simuliidae								+
Thysanoptera			+	+				

#### Testing the synthetic lure in GAS eradication programs

In Mt. Lambert, Trinidad, significantly more GAS (all size classes combined) were killed in plots treated with the synthetic lure and metaldehyde than in control plots (X^2^ = 11.46 (1), P<0.001; Table 6). In Miami, significantly more GAS (all size classes combined) were found near the spot treatments of the synthetic lure in mineral oil emulsion compared to the mineral oil emulsion control (X^2^ = 14.53 (1), P<0.0001; Table 6). Furthermore, as part of the eradication protocol, hand collection of snails and debris removal conducted prior to the application of the lure had removed all visible snails in the study area. Thus, the snails found 12 h after application of the spot treatments had been drawn from the soil and other concealed locations. In addition, only 21% of the 110 plots had snails, indicating a clumped distributions of the snails. In both studies, all size classes of snails were attracted to the lure.

**Table 6:** The total number and size range of giant African snails (*Lissachatina* fulic) found 25 cm from Petri dishes with a cotton wick saturated with liquid metaldehyde or a wick with 0.2% synthetic papaya lure in a 1:1:0.2 emulsion of water, canola oil, and Tween in a field bioassay at Mt. Lambert, Trinidad (n = 25), or 15 cm from 4 ml ground applications of the same blend and a water emulsion control 12 h after application of liquid metaldehyde in Miami, FL, U.S.A. (n = 110).

Treatment	# *Lissachatina fulica*				
	< 24 mm	25–47 mm	>48 mm	Total	Mean(SE)/snails/plot
Mt. Lambert, Trinidad					
Metaldehyde wick	31	23	20	74	3.0 (0.6)
Synthetic lure emulsion wick+ metaldehyde wick	77	77	53	207	8.28 (1.33)
Miami, FL, U.S.A.					
Water emulsion ground application (4 ml)	14	1	0	15	0.14 (0.04)
Synthetic lure emulsion ground application (4 ml)	104	15	2	121	1.1 (0.31)

## Discussion

Attractant lures that draw target species in to traps or toxic baits are important monitoring tools in integrated pest management, and are crucial tools for eradication programs for defining the geographic range that has been infested, to identify habitat preferences, to track the efficacy of control measures, and to assess the outcome of the eradication program [29, 30]. However, their use in gastropod management programs remains relatively unexplored. In this study we showed that a synthetic lure based on the odor of commercial papaya-flavored oil increased the efficacy of pesticide-based methods for controlling GAS by 50–87% compared to controls. The chemicals used to reconstruct the lure were non-toxic and commercially available, often in food grade, making it possible to produce the lure blend with consistent quality, and in any desired amounts required to meet GAS management and eradication program needs.

The synthetic lure drew snails from distances of 35 to 105 cm. In terms of gastropod movement, this is substantial because land snail mobility is generally considered fairly poor [4]. GAS has a limited foraging range, reported to be a few meters [31], and snails may find refuge from management practices in protected areas such as between fences, amongst piles of debris, or under sheds [15]. By using the lure, snails could be drawn to the pesticide-treated areas or into the open where they could be collected. Additionally, this study showed that the snails responded rapidly to the presence of the attractant (< 30 min); thus, the synthetic lure can increase the likelihood that pest gastropods will encounter the pesticide while the pesticide bait is most effective. However, the longevity of the lure was short under field conditions (< 18 h) despite being formulated in an oil emulsion as a means to modulate the release rate of the volatile odors. Further work needs to be done to develop slow-release formulations with longer effective field lifetimes.

One goal of the study was to test different application methods for ease of integration into an eradication program. The lure and oil emulsion applied to a cotton wick was consistently selected over control treatments in laboratory studies as well as in field studies in all 3 geographic locations (Trinidad, Florida, and Hawaii). Snails were also seen to feed for extended periods of time on the wicks (> 60 min) including the 48 h aged wicks, which could increase the duration they would be exposed to pesticides or to surveys conducted by program staff. The effectiveness of adding the lure directly to the metaldehyde spray remains inconclusive. Twice as many snails were found in plots with the lure during the first collection, but a significant difference was not seen in the subsequent collections. This may be due in part to the experimental design. Snails may have crossed control plots inadvertently as they moved toward plots with the lure or they may have entered plots as they returned to daytime refuges. Direct application of the lure emulsion to the ground, as tested in the Florida eradication program, was found to be effective, and provided several advantages. Because the lure formulation was applied directly to the ground as spot treatments, the lures were unaffected by the removal of plant and other debris as part of the eradication protocol, and there were no release devices that needed to be retrieved later. In addition, our results showed that the synthetic lure drew snails into traps, which may be a useful tool, particularly for removing snails from areas that are difficult to access and/or where the presence of pets and farm animals precludes pesticide applications.

The synthetic lure did not attract pets, bees and other protected non-target species. This was an important consideration for use in the Florida GAS eradication program. Metaldehyde-based pesticides that are essential tools for managing GAS [32] are also toxic to wildlife, pets, and livestock [33]. The bran or molasses that is commonly added to the metaldehyde baits to increase its attractiveness to snails and slugs is also attractive and palatable to dogs [5]. As a result, some countries regulate the bait size and application rate as well as requiring that these baits include ingredients to make the bait unpalatable to dogs [34].

Even within gastropods, the synthetic lure showed considerable specificity because it did not attract all slug and snail species present at the study sites. For example, the invasive Cuban slug (*Veronicella cubensis*) and black slug (*Laevicaulis alte*) were not significantly attracted to the lure in any of our studies, nor were they caught in baited Snailer traps. This specificity may prove beneficial in excluding non-target species, and particularly, threatened or endangered gastropods, which may be present in areas where GAS eradication is being attempted. However, the synthetic lure was found to attract another important gastropod pest, *Parmarion martensi*. This species is widely established in Hawaii and is a vector of the nematode *A*. *cantonensis*, the causal agent of angiostrongyliasis [22]. Thus, the synthetic lure may prove to be a useful tool in helping to manage this species.

Food, agricultural waste products, and food grade essences have been tested with varying levels of effectiveness as attractants for GAS [10, 13, 15, 23–25]. Unlike food products, the chemical composition of synthetic lures remains consistent and does not rot, in contrast to aging food baits, which can attract ovipositing flies and vermin. For example, food-based baits have not been used as trap baits in the Florida eradication program because of the high number of empty traps, and the fast, unpleasant decay of the bait [15]. This would not be an issue with the synthetic papaya oil formulation. Additionally, the lure had the combined advantages of significantly enhancing the kill rate of metaldehyde baited plots, of being produced with non-toxic components in defined, controllable, and reproducible ratios, and of having minimal effects on non-target or protected species. When new populations of GAS are discovered, the program now deploys the lure in conjunction with their other control strategies. To our knowledge, this represents the first application of synthetic lures for the management or eradication of any terrestrial gastropod species.

## Supporting information

S1 File Synthetic giant African snail lure laboratory and field evaluation data. (XLSX)
